# Transmission dynamics of hepatitis E among swine: potential impact upon human infection

**DOI:** 10.1186/1746-6148-3-9

**Published:** 2007-05-10

**Authors:** Kunio Satou, Hiroshi Nishiura

**Affiliations:** 1 Department of Epidemiology, National Institute of Animal Health, 3-1-5, Kannnondai, Tsukuba, 305-0856, Japan; 2 Department of Medical Biometry, University of Tübingen, Westbahnhofstr. 55-D, Tübingen, D-72070, Germany; 3 Research Center for Tropical Infectious Diseases, Nagasaki University Institute of Tropical Medicine, Sakamoto 1-12-4, Nagasaki, 852-8523, Japan

## Abstract

**Background:**

Hepatitis E virus (HEV) infection is a zoonosis for which pigs play a role as a reservoir. In Japan, the infection has been enzootic in swine. Clarifying the detailed mechanisms of transmission within farms is required in order to facilitate an understanding of the age-specific patterns of infection, especially just prior to slaughter.

**Results:**

Here we reanalyze a large-scale seroprevalence survey dataset from Japanese pig farms to estimate the force of infection. The forces of infection of swine HEV were estimated to be 3.45 (95% confidence interval: 3.17, 3.75), 2.68 (2.28, 3.14) and 3.11 (2.76, 3.50) [×10^-2 ^per day] in Hokkaido, Honshu and Kyushu, respectively. The estimates with our model assumptions indicated that the average ages at infection ranged from 59.0–67.3 days and that the basic reproduction number, *R*_0_, was in the order of 4.02–5.17. Sensitivity analyses of age-specific incidence at different forces of infection revealed that a decline in the force of infection would elevate the age at infection and could increase the number of virus-excreting pigs at the age of 180 days.

**Conclusion:**

Although our estimates imply that more than 95% of pigs are infected before the age of 150 days, the model shows that a decline in the force of infection could increase the risk of pig-to-human transmission. If the force of infection started to decline, it might be necessary to implement radical countermeasures (e.g. separation of uninfected pigs from infected herds beginning from the end of the suckling stage) to minimize the number of virus-positive pigs at the finishing stage.

## Background

Hepatitis E virus (HEV) is a positive-strand RNA virus without an envelope, which is classified as a member of the genus *Hepevirus *in the family *Hepeviridae *[1,2]. The virus is distributed worldwide, especially in the tropical and subtropical regions of Asia, Africa and Latin America, causing acute hepatitis in humans and is thus an important public health problem [3]. HEV infection is a zoonosis mainly seen in humans and pigs [4-8]. In addition to the maintenance of the virus in swine as a reservoir [9], the infection is also seen in other primates [10-12]. The virus is mainly transmitted via fecal-oral routes among swine [13,14]. Whereas humans are also enterically infected mainly through contaminated foods, a water-borne outbreak can be caused if drinking water is contaminated with feces containing the virus [15].

HEV infection in humans is seen not only in developing countries but also in industrialized countries where sporadic cases of infection have been reported [16]. In particular, sporadic cases in various places and settings have been reported in Japan [16-23]. Whereas deer have been suggested to be a source of human infection [17,18], ingestion of uncooked liver from wild boar is also frequently reported as the cause of infection [19-23]. In addition to the habitual consumption of porcine liver in Japan, it is important to note that the HEV infection is enzootic in swine, facilitating the frequent occurrence of pig-to-human transmission [12,24,25]. Seroprevalence surveys in other industrialized countries have also demonstrated the occurrence of virus transmission in swine [14,26-30]. Although it is still yet to be fully clarified, pigs are believed to be the natural host for the virus [5,10,16].

With these points in mind, it is essential to clarify the detailed mechanisms of HEV transmission in swine. For example, it would be very useful to know the average age of individuals acquiring infection in enzootic areas and the age-specific incidence, especially just prior to slaughter. Moreover, to identify effective control measures on the farm (e.g., potential vaccination strategy [31]), it would be necessary to quantify a key parameter of the transmission, the basic reproduction number, *R*_0_, defined as the average number of secondary cases arising from a single primary case in a fully susceptible population. *R*_0 _gives an indication of the transmission potential, and thus, is one of the most important epidemiologic determinants [32,33]. For example, in a randomly mixing population, a critical coverage of vaccination to eradicate a disease, *p*_c_, can be derived by using *R*_0_; *p*_c _> 1-1/*R*_0 _[34]. In enzootic areas, an estimate of *R*_0 _can be approximately obtained by estimating the force of infection (i.e. the rate at which susceptible individuals become infected), *λ*, which is derived from age-specific seroprevalence data. For nearly half a century, a catalytic model, the most classic type of force of infection model [35], has been applied to seroprevalence and incidence data and various extensions have been proposed [36-40].

The aim of this paper was to assess the transmission potential of HEV infection in swine using seroprevalence survey data from Japan. To clarify the age-specific mechanisms of transmission in swine raised for human consumption, published data [24] on age-specific seroprevalence on pig farms was re-examined. With respect to data from farms where pigs are slaughtered immediately after the age of 180 days, there are two specific characteristics which required close epidemiologic attention: (1) since the pigs at the suckling stage (i.e., younger than 30 days of age) are raised in separate housing from those in later stages, and due partly to a very short-lived maternal antibody, those younger than 30 days are not exposed to infection [24,25], and (2) compared to the demographic time scale (i.e. life expectancy being 180 days), the time required for seroconversion is relatively long and is not insignificant [9]. Thus, we propose our original modeling strategy assuming that exposure starts at the age of 30 days and combining the explicit estimate of the time required for seroconversion with a simple model of the force of infection.

## Results

### Time required for seroconversion

**Figure 1:**
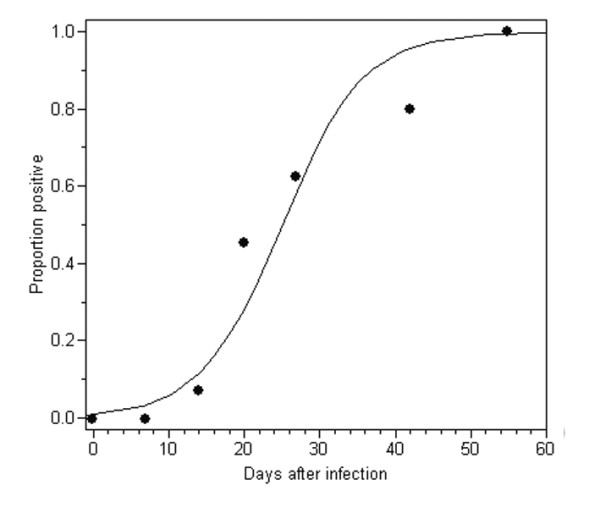
**Observed and predicted time required for seroconversion of anti-HEV antibody**. Logit model (line) was applied to the observed cumulative distribution of the time required for seroconversion (dot), which revealed a sigmoid pattern. Data source: ref. [9].

A simple logit model was applied to the cumulative distribution of the time required for seroconversion of anti-HEV antibody based on inoculation experiments using swine HEV [9]. Figure 1 compares the observed and predicted proportion of seroconversion with time post-inoculation. The maximum likelihood estimate of the median time required for seroconversion was 25.0 (95% confidence interval (CI): 20.9, 31.3) days. The coefficient of the time after inoculation (for the logit model) was estimated as 0.19 (95% CI: 0.11, 0.30). The *χ*^2 ^goodness-of-fit test did not reveal significant deviations between observed and predicted frequencies (*χ*^2^_5 _= 2.31, p = 0.81).

### Estimates of the force of infection

**Figure 2:**
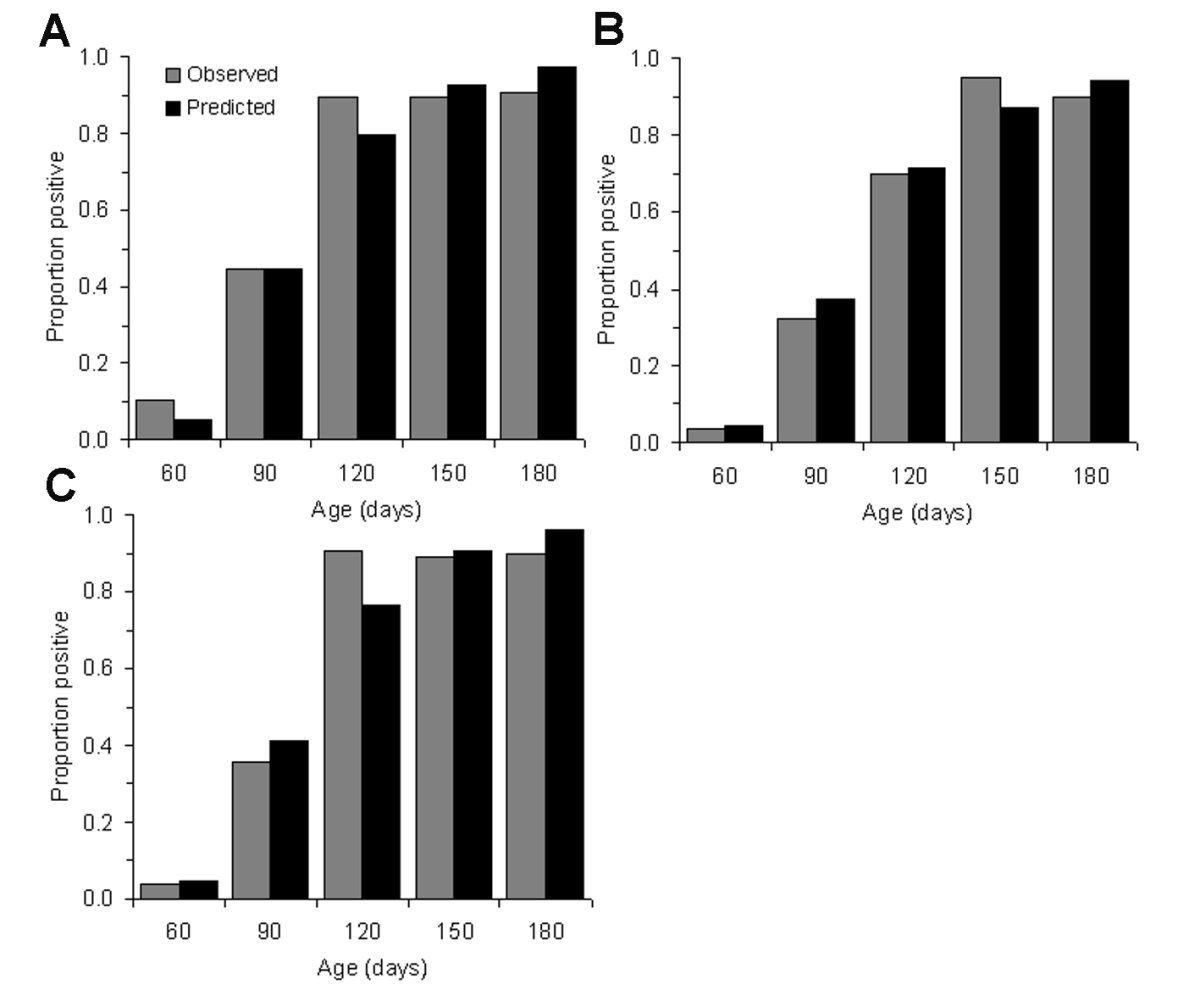
**Observed and predicted age-specific seroprevalence against swine hepatitis E virus in Japan**. Observed (gray bar) and predicted (black) seroprevalence are compared. Three discrete geographic areas, Hokkaido (**A**), Honshu (**B**) and Kyushu (**C**), are modeled separately. Data source: ref. [24].

The total sample sizes of seroprevalence surveys were 1400, 400 and 700 for Hokkaido, Honshu and Kyushu, respectively [24]. The maximum likelihood estimates of the force of infection, *λ*, (and the corresponding 95% CI) of swine HEV for the three different geographic locations in Japan were 3.45 (3.17, 3.75), 2.68 (2.28, 3.14) and 3.11 (2.76, 3.50) [×10^-2 ^per day]. Expected values of *λ *ranging from 2.68–3.45 ×10^-2 ^day^-1 ^indicate that the average time of infection ranged from 29.0–37.3 days after the age of 30 days when individuals were first exposed to the risk of infection (i.e. average age at infection was 59.0–67.3 days). Observed and predicted age-specific seroprevalence are compared in Figure 2, confirming good overall agreement of the model with the data. Although a significant deviation was seen in Hokkaido (*χ*^2^_4 _= 16.71, p < 0.01), this was not the case for Honshu (*χ*^2^_4 _= 1.49, p = 0.89) or Kyushu (*χ*^2^_4 _= 6.34, p = 0.17).

The population structure on pig farms in Japan is specific in that individuals are slaughtered immediately after the age of 180 days (i.e., 150 days after pigs are first exposed to the risk of infection). This satisfied a reasonable approximation of a simple age-specific survivorship function, referred to as Type I survivorship [32], to the data:

μ(a)=0,fora<180μ(a)=∞,fora≥180
 MathType@MTEF@5@5@+=feaafiart1ev1aaatCvAUfKttLearuWrP9MDH5MBPbIqV92AaeXatLxBI9gBaebbnrfifHhDYfgasaacH8akY=wiFfYdH8Gipec8Eeeu0xXdbba9frFj0=OqFfea0dXdd9vqai=hGuQ8kuc9pgc9s8qqaq=dirpe0xb9q8qiLsFr0=vr0=vr0dc8meaabaqaciaacaGaaeqabaqabeGadaaakqaabeqaaGGaciab=X7aTjabcIcaOiabdggaHjabcMcaPiabg2da9iabicdaWiabcYcaSGGabiab+bcaGiab+bcaGiabdAgaMjabd+gaVjabdkhaYjab+bcaGiab+bcaGiabdggaHjabgYda8iabigdaXiabiIda4iabicdaWaqaaiab=X7aTjabcIcaOiabdggaHjabcMcaPiabg2da9iabg6HiLkabcYcaSiab+bcaGiab+bcaGiabdAgaMjabd+gaVjabdkhaYjab+bcaGiab+bcaGiabdggaHjabgwMiZkabigdaXiabiIda4iabicdaWaaaaa@55DB@

where *μ*(*a*) denotes age-specific mortality rate at age *a*. The basic reproduction number, *R*_0_, is approximated as the product of the force of infection, *λ*, and the time from first exposure to death, *L *(= 150 days) [32]:

*R*_0 _≈ *λL*

Thus, *R*_0 _(and the 95% CI) was estimated to be 5.17 (4.76, 5.62), 4.02 (3.43, 4.71) and 4.66 (4.13, 5.25) for Hokkaido, Honshu and Kyushu, respectively.

### Age-specific incidence at different forces of infection

**Figure 3:**
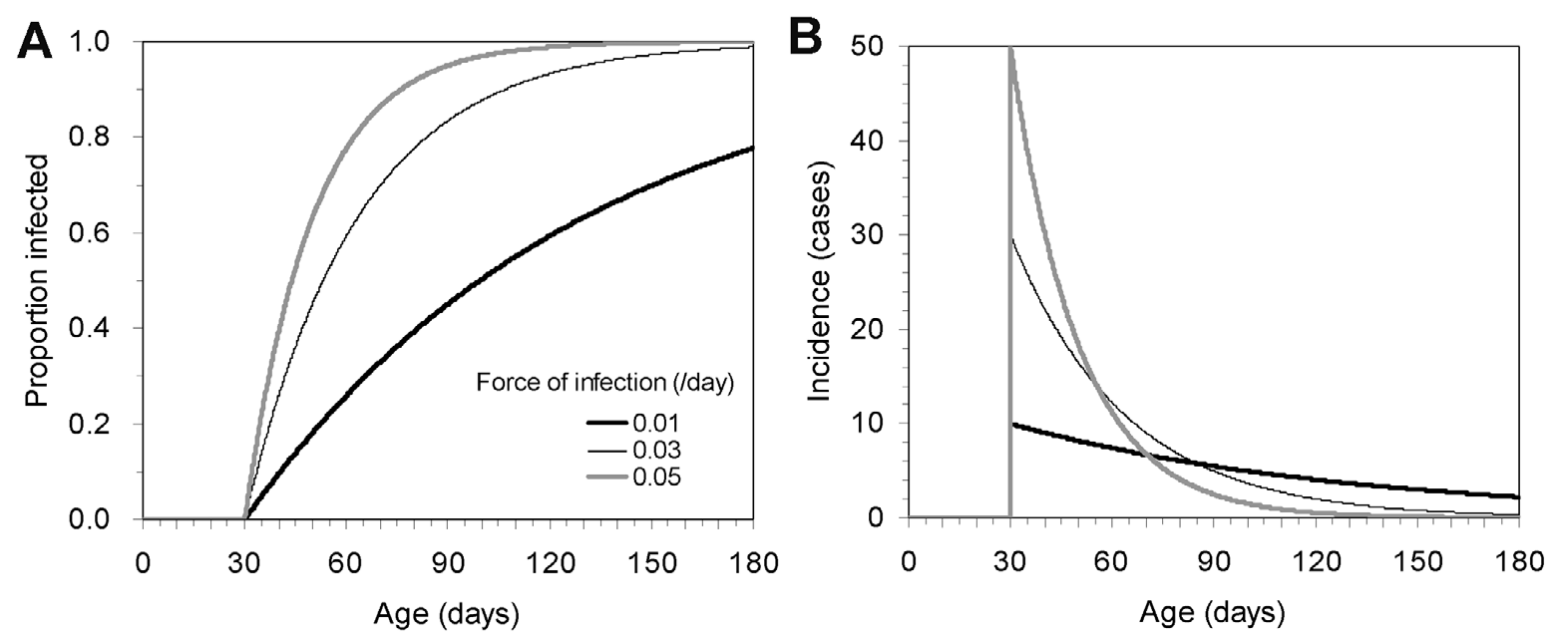
**Cumulative frequency of infection and age-specific incidence at different forces of infection**. **A**. Cumulative frequencies of HEV infection and **B**. age-specific incidence elicited by different forces of infection are compared. Assumed values for the forces of infection were 0.01 (thick black), 0.03 (thin black) and 0.05 (thick gray) days^-1^. See eqs. 6 and 7 for details of the model.

Figure 3A shows the cumulative proportion of infected individuals according to our simple assumptions with different forces of infection, *λ*, being 0.01, 0.03 and 0.05 (day^-1^). Accordingly, the average ages at infection, *A *(= 30 + 1/*λ*), are 130, 63 and 50 days, respectively. Using this range of *λ*, 0.03 (0.01, 0.05), it is predicted that 83.5 % (45.1, 95.0) of the herd would be infected at the age of 90 days, 93.3 % (59.3, 98.9) at 120 days and 97.3 % (69.9, 99.8) at 150 days. Based on the same assumptions, Figure 3B shows the age-specific absolute incidence (i.e. the number of newly infected individuals) at each given age in a hypothetical herd with a population size of 1000. Despite a few rough assumptions, the figure indicates that average age at infection directly influences the age-specific patterns of HEV incidence, enabling the prediction of incidence shortly before the age of finishing (i.e. 180 days). According to the above range of *λ*, the model predicts that 2.1 (4.1, 0.6) individuals would be newly infected at the age of 120 days, 0.8 (3.0, 0.1) at 150 days and 0.3 (2.3, 0.0) at 180 days. In other words, the smaller the force of infection is, higher the probability that virus excretion would occur at the finishing stage.

## Discussion

This study estimated the force of infection of swine HEV for three geographic locations in Japan. For the estimation, we incorporated two realistic aspects of swine HEV transmission: (1) no exposure during the suckling stage and (2) time delay of seroconversion after exposure to the virus. As a result, the force of infection was estimated to be approximately 0.03 day^-1 ^implying that the average age at infection is 63 days after birth. According to the estimates, the basic reproduction number, *R*_0_, was in the order of 4–5, which is relatively high compared to other diseases [32,41]. To the best of our knowledge, this study is the first to quantify the transmission potential of swine HEV infection. Although the model needs a few rough assumptions, and despite limited precision of the observed data (i.e. seroprevalence data was only collected monthly), our model successfully provides similar estimates of *λ *for 3 discrete locations. Except for a slight deviation seen in Hokkaido where the samples were taken from numerous sub-regions in the large prefecture, the model adequately explained the basic aspects of the age-specific pattern of HEV seroprevalence in swine. Estimated force of infection was highest in Hokkaido, the northernmost prefecture, while Honshu revealed the lowest estimate. The force of infection depends on various factors influencing transmission (e.g. biological, environmental and demographic factors). In particular, as the disease is transmitted through virus contamination (i.e. fecal-oral route), breeding methods and other determinants affecting exposure are likely to influence the age-specific patterns of prevalence. Whereas the farms in Hokkaido were partly infested with both genotypes III and IV, only genotype III was observed in the other two regions. However, since these two genotypes are immunologically crossreactive each other [5,42], these could not be separately evaluated without detailed information with respect to differences in natural history and immune reaction.

There are two practical implications from our exercise. First, estimation of the force of infection permitted clarification of the average age at infection (being 63 days). Although our model did not allow more detailed age- and time-specificity of the force of infection to be derived due to limited data [37-40], knowing the average age at infection enables clarification of the age-specific incidence of infection (as shown in Figure 3), thereby providing a reasonable assessment of the risk of HEV excretion in slaughtered pigs. According to rigorous inoculation experiments [9,13,43], swine HEV RNA can be detected in the liver, feces, bile and other parts of the body as long as 30 days post-inoculation. In enzootic areas, therefore, pigs should ideally be infected sufficiently far in advance of reaching 150 days of age, so that the probability of virus excretion will be extremely low at 180 days. Although our estimates of the force of infection in Japan imply that the majority of individuals (i.e., more than 95%) are infected before the age of 150 days, it should be noted that any future decline in the force of infection would increase the number of virus-positive pigs at the age of 180 days. Thus, most importantly, it must be remembered that a slight decline in the force of infection could elevate the age at infection and increase the risk of pig-to-human transmission. In addition to consumption of contaminated pork by the general public, the increased risk of infection could also be a particularly risk for veterinarians and boar meat processing workers [44,45]. If the force of infection is naturally reduced on the farm, this could necessitate radical control measures to minimize the number of virus-excreting pigs at the finishing stage and to eliminate the transmission from the farm. Since the population dynamics model can account for more detailed mechanisms of transmission [46-48], further explicit clarification on this point is a subject of our further studies. Although the time required for seroconversion may be slightly underestimated (because of the estimation using intravenous inoculation rather than that through oral routes), this underestimation would only result in slight underestimation of the force of infection, and thus, the above qualitative discussion of the results and their implications is still valid.

Second, the critical coverage of vaccination required for eradication, *p*_c_, is obtained from *R*_0_, using *p*_c _> 1-1/*R*_0 _[34]. Although vaccines are currently under development [31], our estimate of *R*_0_, ranging from 4.02–5.17, suggests that the HEV transmission on the farm could be prevented if more than 75.1–80.7 % of the pigs were successfully immunized. However, since HEV infection in man is likely to result in asymptomatic or mild disease [3,16,49], and because pig-to-human transmission could be partly prevented by dietary changes of humans (i.e. avoiding consumption of fresh liver), potential future vaccination policies for swine need to take account of cost-benefit analyses and the biological feasibility of elimination. For example, the maintenance of the virus by other primates could prevent the elimination of virus transmission in swine [3,10]. Rather, if it becomes necessary to implement radical control measures, it may be more realistic and less costly to control the transmission within a herd at specific stages; considering that more than four-fifths of infection had happened between the ages of 30 and 90 days, temporal separation of uninfected young pigs from infected herds beginning from the end of suckling stage (e.g. for a certain time period, breed the individuals in a new house) could limit the chance of continued transmission. In this case, tight management of newly-built pig farms (i.e. prevention of contamination from other locations and animals) combined with the possibility of vaccination in the future might be necessary to reduce transmission within the herd.

## Conclusion

The force of infection of swine HEV was estimated from three discrete geographic locations in Japan using age-specific seroprevalence data. The estimates ranged from 2.68–3.45 ×10^-2 ^(day^-1^), indicating that *R*_0 _ranges from 4.02–5.17. The estimates permitted a reasonable prediction of the age-specific incidence including that at the finishing stage. Although our estimates of the force of infection imply that more than 95% are infected before the age of 150 days and the probability of virus-excretion is small at 180 days, the model suggests that a decline in the force of infection could elevate the average age at infection and increase the risk of pig-to-human transmission. If the force of infection started to decline, it might be necessary to implement radical countermeasures (e.g. separation of uninfected pigs from infected herds beginning from the end of the suckling stage) to minimize the number of virus-positive pigs at the finishing stage. As this study showed a reasonable estimation in Japan which is an enzootic area for swine HEV infection, similar seroprevalence survey would be extremely useful to decipher the mechanisms of transmission. Thus, seroepidemiologic studies of swine, human and other animals with time, space and age as well as among specific groups [44,45] could shed further light on the transmission dynamics of HEV.

## Methods

### Data

**Figure 4:**
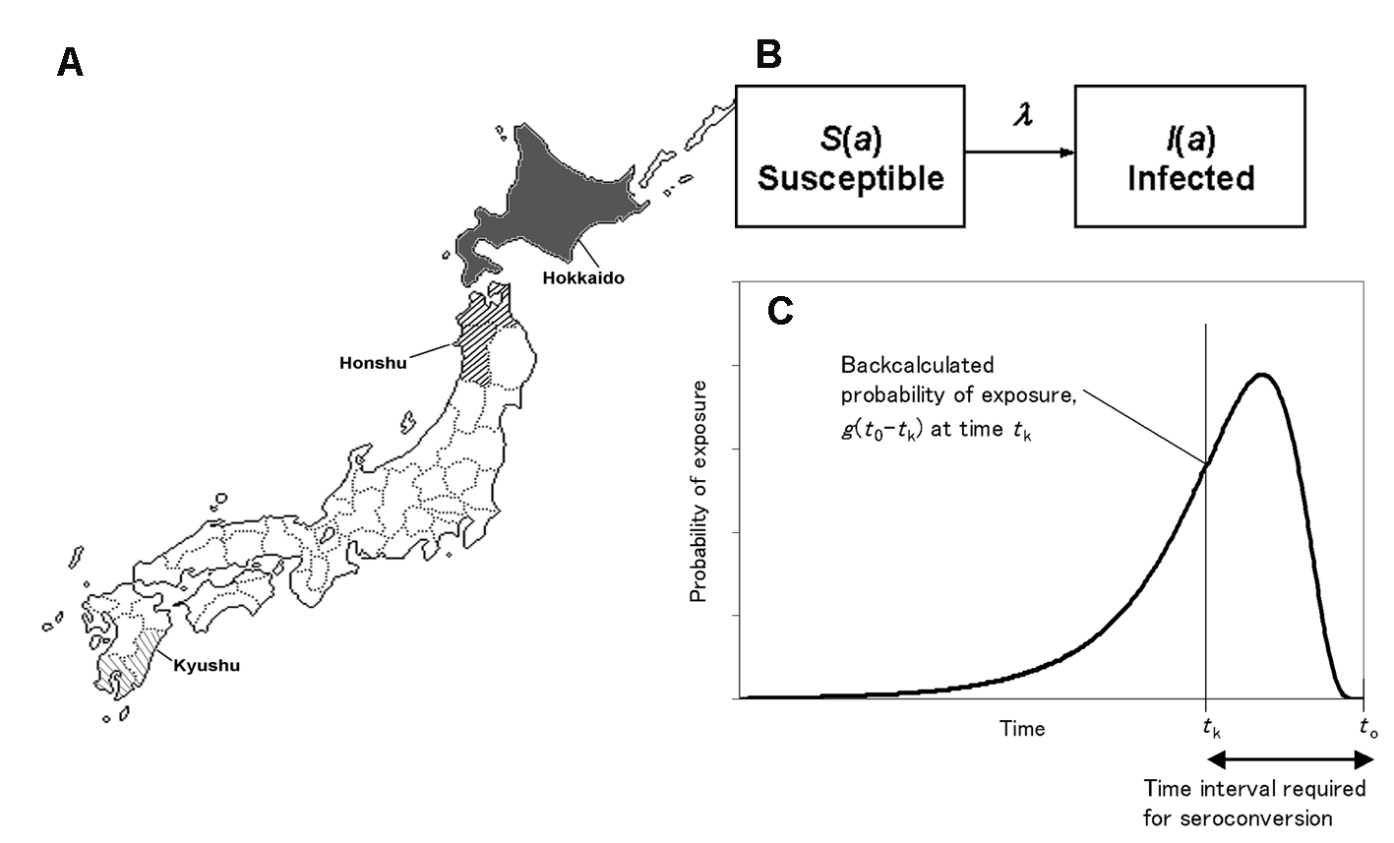
**Estimation of the force of infection of hepatitis E virus in Japan**. **A**. The map of three geographic locations in Japan (drawn by the authors). **B**. Compartment of the catalytic model. Susceptibles at age *a*, *S*(*a*), are infected at a rate *λ *and then enter into the compartment, infected, *I*(*a*). **C**. Schematic illustration of the time delay to seroconvert. If the time of seroconversion *t*_0 _and possible time of exposure *t*_k _are given, probability of exposure at time *t*_k _can be extracted by *g*(*t*_0_-*t*_k_), where g(*t*) is the probability density of the time required for seroconversion at *t *days after infection. See eqs. 9 and 10 for statistical details.

To estimate the force of infection, this study used two published datasets: (1) an experimental inoculation study of swine HEV [9], and (2) a large-scale seroprevalence survey in Japan [24]. The experimental study recorded the time required for seroconversion of anti-HEV antibody following inoculation. The seroepidemiologic study consisted of a survey of pig farms in three discrete geographic locations (Hokkaido, Honshu and Kyushu; see Figure 4A). Anti-HEV IgG antibodies of 2500 pigs were measured by age group. The original study examined the age-specific seroprevalence by month, i.e., pigs at 60, 90, 120, 150 and 180 days of age (allowing ± 5 days variation for each) were sampled [24]. We used this data because the detailed breeding methods of the piggery in Japan were known and the sample size was sufficiently large to allow statistical analysis. On the farm, a proportion of the suckling herd has a very short-lived maternal antibody (Tsunemitsu H, personal communication) and is not exposed to infection due to separate housing during this stage. A seroepidemiologic study and an isolation study of HEV RNA during natural infection in other countries also roughly satisfied this assumption: the number of seropositive pigs was negligible at the age of 30 days on several farms [50] and HEV RNA started to be detected at the age of 30 days [51]. Since the pigs thereafter entered into weaning, growing and fattening stages, we assume that the risk of exposure starts at the age of 30 days. The pigs are slaughtered immediately after the age of 180 days.

### Estimation of the time required for seroconversion

Cumulative distribution of the time required for seroconversion, *G*(*t*) at *t *days post-inoculation was approximated by a simple logit model:

G(t)=exp⁡(l)1+exp⁡(l)
 MathType@MTEF@5@5@+=feaafiart1ev1aaatCvAUfKttLearuWrP9MDH5MBPbIqV92AaeXatLxBI9gBaebbnrfifHhDYfgasaacH8akY=wiFfYdH8Gipec8Eeeu0xXdbba9frFj0=OqFfea0dXdd9vqai=hGuQ8kuc9pgc9s8qqaq=dirpe0xb9q8qiLsFr0=vr0=vr0dc8meaabaqaciaacaGaaeqabaqabeGadaaakeaacqWGhbWrcqGGOaakcqWG0baDcqGGPaqkcqGH9aqpdaWcaaqaaiGbcwgaLjabcIha4jabcchaWjabcIcaOiabdYgaSjabcMcaPaqaaiabigdaXiabgUcaRiGbcwgaLjabcIha4jabcchaWjabcIcaOiabdYgaSjabcMcaPaaaaaa@425C@

where *l *is a linear predictor given by:

*l *= *b*(*t *- *t*_*m*_)

where *b *is the coefficient of the time since inoculation and *t*_m _is the median time required for seroconversion. In order to apply a logistic curve to the cumulative distribution, the model has to satisfy *G*(0) ≈ 0 and *G*(∞) = 1. The maximum likelihood estimates of *b *and *t*_m _were obtained by minimizing the binomial deviance of the model from the observed data. The 95% CI were determined by using the profile likelihood.

### Force of infection

For simplicity and due to limited data availability, we assumed that the force of infection, *λ*, was independent of time (i.e. endemic equilibrium). Furthermore, except for an assumption that no exposure occured during the suckling stage (i.e. until the age of 30 days), *λ *was also assumed to be age-independent. Figure 4B shows a schematic illustration of the model. We denote the proportions of susceptible and infected individuals at age *a *days (for *a *≥ 30) by *S*(*a*) and *I*(*a*), respectively. With an initial condition, *S*(30) = 1 and *I*(30) = 0, the model is given by the following ordinal differential equations:

dS(a)da=−λS(a)dI(a)da=λS(a)
 MathType@MTEF@5@5@+=feaafiart1ev1aaatCvAUfKttLearuWrP9MDH5MBPbIqV92AaeXatLxBI9gBaebbnrfifHhDYfgasaacH8akY=wiFfYdH8Gipec8Eeeu0xXdbba9frFj0=OqFfea0dXdd9vqai=hGuQ8kuc9pgc9s8qqaq=dirpe0xb9q8qiLsFr0=vr0=vr0dc8meaabaqaciaacaGaaeqabaqabeGadaaakqaabeqaamaalaaabaGaemizaqMaem4uamLaeiikaGIaemyyaeMaeiykaKcabaGaemizaqMaemyyaegaaiabg2da9iabgkHiTGGaciab=T7aSjabdofatjabcIcaOiabdggaHjabcMcaPaqaamaalaaabaGaemizaqMaemysaKKaeiikaGIaemyyaeMaeiykaKcabaGaemizaqMaemyyaegaaiabg2da9iab=T7aSjabdofatjabcIcaOiabdggaHjabcMcaPaaaaa@4BAC@

for *a *≥ 30. The analytical solution is given by:

*I*(*a*) = 1-exp{-*λ*(*a*-30)}

for *a *≥ 30. Under the same assumption, age-specific incidence, *C*(*a*), at age *a*, is given by product of *λ *and *S*(*a*):

C(a)=0fora<30C(a)=λexp⁡{−λ(a−30)}fora≥30
 MathType@MTEF@5@5@+=feaafiart1ev1aaatCvAUfKttLearuWrP9MDH5MBPbIqV92AaeXatLxBI9gBaebbnrfifHhDYfgasaacH8akY=wiFfYdH8Gipec8Eeeu0xXdbba9frFj0=OqFfea0dXdd9vqai=hGuQ8kuc9pgc9s8qqaq=dirpe0xb9q8qiLsFr0=vr0=vr0dc8meaabaqaciaacaGaaeqabaqabeGadaaakeaafaqaaeGacaaabaGaem4qamKaeiikaGIaemyyaeMaeiykaKIaeyypa0JaeGimaadabaGaemOzayMaem4Ba8MaemOCaihcceGae8hiaaIae8hiaaIaemyyaeMaeyipaWJaeG4mamJaeGimaadabaGaem4qamKaeiikaGIaemyyaeMaeiykaKIaeyypa0dcciGae43UdWMagiyzauMaeiiEaGNaeiiCaa3aaiWaaeaacqGHsislcqGF7oaBcqGGOaakcqWGHbqycqGHsislcqaIZaWmcqaIWaamcqGGPaqkaiaawUhacaGL9baaaeaacqWGMbGzcqWGVbWBcqWGYbGCcqWFGaaicqWFGaaicqWGHbqycqGHLjYScqaIZaWmcqaIWaamaaaaaa@5CE8@

In addition to the estimation of *λ*, we examined the sensitivity of *I*(*a*) and *C*(*a*) to the different values of *λ *to explore the age-specific patterns and clarify the practical implications of *λ *to farm management.

### Convolution equation and maximum likelihood estimation

Since all individuals are slaughtered immediately after the age of 180 days, the time delay from infection to seroconversion was thought to be non-negligible. That is, the age-specific seroprevalence data (at age *a *days) does not directly reflect all of those who were infected until age *a*, *I*(*a*), but rather indicates those infected until the age which is smaller than *a*. Thus, to account for the delay, we used probability density of the time to seroconvert (Figure 4C) in addition to the model of the cumulative distribution of HEV infection (Eq. 6). Considering the time-scale of the distribution of time to seroconvert (Figure 1) and the cumulative incidence up to the age of 60 days (Figure 2), we assumed that those seropositive at the age of 60 days approximately reflected those who were infected until the age of 45 days. Under these assumptions, the age-specific incidence density, *f*_i_, whose infection is reflected as seropositive at *i *months after starting to be exposed (i.e., *a *= 30(*i *+ 1) days) is assumed to be:

fi=I(45)fori=1fi=I(30i+15)−I(30i−15)for2≤i≤5
 MathType@MTEF@5@5@+=feaafiart1ev1aaatCvAUfKttLearuWrP9MDH5MBPbIqV92AaeXatLxBI9gBaebbnrfifHhDYfgasaacH8akY=wiFfYdH8Gipec8Eeeu0xXdbba9frFj0=OqFfea0dXdd9vqai=hGuQ8kuc9pgc9s8qqaq=dirpe0xb9q8qiLsFr0=vr0=vr0dc8meaabaqaciaacaGaaeqabaqabeGadaaakeaafaqaaeGacaaabaGaemOzay2aaSbaaSqaaiabdMgaPbqabaGccqGH9aqpcqWGjbqscqGGOaakcqaI0aancqaI1aqncqGGPaqkaeaacqWGMbGzcqWGVbWBcqWGYbGCiiqacqWFGaaicqWFGaaicqWGPbqAcqGH9aqpcqaIXaqmaeaacqWGMbGzdaWgaaWcbaGaemyAaKgabeaakiabg2da9iabdMeajjabcIcaOiabiodaZiabicdaWiabdMgaPjabgUcaRiabigdaXiabiwda1iabcMcaPiabgkHiTiabdMeajjabcIcaOiabiodaZiabicdaWiabdMgaPjabgkHiTiabigdaXiabiwda1iabcMcaPaqaaiabdAgaMjabd+gaVjabdkhaYjab=bcaGiab=bcaGiabikdaYiabgsMiJkabdMgaPjabgsMiJkabiwda1aaaaaa@615F@

Using the same mid-point of the time-interval, the probability density of time to seroconvert, *g*_j_, at *j *months after infection was also approximated by using *G*(*t*):

$$\begin{array}{ll} g_{j}=G(15) & forj=1\\ g_{j}=G(30j-15)-G(30j-45) & for2\leq j\leq 5 \end{array}$$

The density of those who newly showed seropositive results at the age of 60 days (i.e., at *i *= 1 month), *k*_1_, is given by *k*_1 _= *f*_1_g_1_. In the same way, the densities at the ages of 90 and 120 days, *k*_2 _and *k*_3_, are given by *k*_2 _= *f*_2_g_1 _+ *f*_1_g_2 _and *k*_3 _= *f*_3_g_1 _+ *f*_2_g_2 _+ *f*_1_g_3_. This can be generalized by using the following convolution equation [52-54]:

ki=∑j=1ifi−j+1gj
 MathType@MTEF@5@5@+=feaafiart1ev1aaatCvAUfKttLearuWrP9MDH5MBPbIqV92AaeXatLxBI9gBaebbnrfifHhDYfgasaacH8akY=wiFfYdH8Gipec8Eeeu0xXdbba9frFj0=OqFfea0dXdd9vqai=hGuQ8kuc9pgc9s8qqaq=dirpe0xb9q8qiLsFr0=vr0=vr0dc8meaabaqaciaacaGaaeqabaqabeGadaaakeaacqWGRbWAdaWgaaWcbaGaemyAaKgabeaakiabg2da9maaqahabaGaemOzay2aaSbaaSqaaiabdMgaPjabgkHiTiabdQgaQjabgUcaRiabigdaXaqabaGccqWGNbWzdaWgaaWcbaGaemOAaOgabeaaaeaacqWGQbGAcqGH9aqpcqaIXaqmaeaacqWGPbqAa0GaeyyeIuoaaaa@4169@

Since the observed seroprevalence data shows the cumulative distribution of those seroconverted after infection until month *i*, *K*_i_, which is given by Ki=∑m=1ikm
 MathType@MTEF@5@5@+=feaafiart1ev1aaatCvAUfKttLearuWrP9MDH5MBPbIqV92AaeXatLxBI9gBaebbnrfifHhDYfgasaacH8akY=wiFfYdH8Gipec8Eeeu0xXdbba9frFj0=OqFfea0dXdd9vqai=hGuQ8kuc9pgc9s8qqaq=dirpe0xb9q8qiLsFr0=vr0=vr0dc8meaabaqaciaacaGaaeqabaqabeGadaaakeaacqWGlbWsdaWgaaWcbaGaemyAaKgabeaakiabg2da9maaqahabaGaem4AaS2aaSbaaSqaaiabd2gaTbqabaaabaGaemyBa0Maeyypa0JaeGymaedabaGaemyAaKganiabggHiLdaaaa@3A3B@, we get:

Ki=∑j=1ifi−j+1Gj
 MathType@MTEF@5@5@+=feaafiart1ev1aaatCvAUfKttLearuWrP9MDH5MBPbIqV92AaeXatLxBI9gBaebbnrfifHhDYfgasaacH8akY=wiFfYdH8Gipec8Eeeu0xXdbba9frFj0=OqFfea0dXdd9vqai=hGuQ8kuc9pgc9s8qqaq=dirpe0xb9q8qiLsFr0=vr0=vr0dc8meaabaqaciaacaGaaeqabaqabeGadaaakeaacqWGlbWsdaWgaaWcbaGaemyAaKgabeaakiabg2da9maaqahabaGaemOzay2aaSbaaSqaaiabdMgaPjabgkHiTiabdQgaQjabgUcaRiabigdaXaqabaGccqWGhbWrdaWgaaWcbaGaemOAaOgabeaaaeaacqWGQbGAcqGH9aqpcqaIXaqmaeaacqWGPbqAa0GaeyyeIuoaaaa@40E9@

where Gi=∑m=1igm
 MathType@MTEF@5@5@+=feaafiart1ev1aaatCvAUfKttLearuWrP9MDH5MBPbIqV92AaeXatLxBI9gBaebbnrfifHhDYfgasaacH8akY=wiFfYdH8Gipec8Eeeu0xXdbba9frFj0=OqFfea0dXdd9vqai=hGuQ8kuc9pgc9s8qqaq=dirpe0xb9q8qiLsFr0=vr0=vr0dc8meaabaqaciaacaGaaeqabaqabeGadaaakeaacqWGhbWrdaWgaaWcbaGaemyAaKgabeaakiabg2da9maaqahabaGaem4zaC2aaSbaaSqaaiabd2gaTbqabaaabaGaemyBa0Maeyypa0JaeGymaedabaGaemyAaKganiabggHiLdaaaa@3A2B@. Suppose that there were *N*_i _pigs who had been seropositive until month *i *(where *i *= *a*/30 - 1) and *M*_i _who had not, the likelihood function is

L=∏h=15KhNh(1−Kh)Mh
 MathType@MTEF@5@5@+=feaafiart1ev1aaatCvAUfKttLearuWrP9MDH5MBPbIqV92AaeXatLxBI9gBaebbnrfifHhDYfgasaacH8akY=wiFfYdH8Gipec8Eeeu0xXdbba9frFj0=OqFfea0dXdd9vqai=hGuQ8kuc9pgc9s8qqaq=dirpe0xb9q8qiLsFr0=vr0=vr0dc8meaabaqaciaacaGaaeqabaqabeGadaaakeaacqWGmbatcqGH9aqpdaqeWbqaaiabdUealnaaDaaaleaacqWGObaAaeaacqWGobGtdaWgaaadbaGaemiAaGgabeaaaaGccqGGOaakcqaIXaqmcqGHsislcqWGlbWsdaWgaaWcbaGaemiAaGgabeaakiabcMcaPmaaCaaaleqabaGaemyta00aaSbaaWqaaiabdIgaObqabaaaaaWcbaGaemiAaGMaeyypa0JaeGymaedabaGaeGynaudaniabg+Givdaaaa@43B8@

The maximum likelihood estimate of *λ *is obtained by minimizing the negative logarithm of Eq. 12. The 95% CI were derived from the profile likelihood. All statistical data were analyzed using the statistical software JMP IN ver. 5.1 (SAS Institute Inc., Cary, NC).

## Abbreviations

HEV – Hepatitis E virus

CI – Confidence interval

## Competing interests

The author(s) declare that they have no competing interests.

## Authors' contributions

KS and HN carried out paper reviews, proposed the study, performed statistical analyses and drafted the manuscript together. The authors have read and approved the final manuscript.
